# Rheumatoid arthritis synovium contains plasmacytoid dendritic cells

**DOI:** 10.1186/ar1467

**Published:** 2005-01-11

**Authors:** Lois L Cavanagh, Amanda Boyce, Louise Smith, Jagadish Padmanabha, Luis Filgueira, Peter Pietschmann, Ranjeny Thomas

**Affiliations:** 1 Centre for Immunology and Cancer Research, University of Queensland, Princess Alexandra Hospital, Brisbane, Australia; 2 Institute of Anatomy, University Irchel-Zurich, Zurich, Switzerland; 3 Department of General and Experimental Pathology, University of Vienna, Vienna, Austria

**Keywords:** dendritic cells, plasmacytoid, rheumatoid arthritis, TNF

## Abstract

We have previously described enrichment of antigen-presenting HLA-DR^+ ^nuclear RelB^+ ^dendritic cells (DCs) in rheumatoid arthritis (RA) synovium. CD123^+^HLA-DR^+ ^plasmacytoid DCs (pDCs) and their precursors have been identified in human peripheral blood (PB), lymphoid tissue, and some inflamed tissues. We hypothesized recruitment of pDCs into the inflamed RA synovial environment and their contribution as antigen-presenting cells (APCs) and inflammatory cells in RA. CD11c^+ ^myeloid DCs and CD123^+ ^pDCs were compared in normal and RA PB, synovial fluid (SF), and synovial tissue by flow cytometry, immunohistochemistry, and electron microscopy and were sorted for functional studies. Nuclear RelB^-^CD123^+ ^DCs were located in perivascular regions of RA, in a similar frequency to nuclear RelB^+^CD123^- ^DCs, but not normal synovial tissue sublining. Apart from higher expression of HLA-DR, the numbers and phenotypes of SF pDCs were similar to those of normal PB pDCs. While the APC function of PB pDCs was less efficient than that of PB myeloid DCs, RA SF pDCs efficiently activated resting allogeneic PB T cells, and high levels of IFN-γ, IL-10, and tumor necrosis factor α were produced in response to incubation of allogeneic T cells with either type of SF DCs. Thus, pDCs are recruited to RA synovial tissue and comprise an APC population distinct from the previously described nuclear RelB^+ ^synovial DCs. pDCs may contribute significantly to the local inflammatory environment.

## Introduction

Plasmacytoid dendritic cells (pDCs) are a distinct population of antigen-presenting cells (APCs) with the capacity for potent antigen-presenting function and production of large amounts of cytokines, including tumor necrosis factor (TNF)-α and IFN-α. Human pDCs can be identified by cell-surface expression of MHC molecules, the α-chain of the IL-3 receptor (CD123), and the presence of blood dendritic-cell (DC) antigens known as BDCA2 and BDCA4 in a proportion of cells [1]. In comparison with CD11c^+ ^myeloid DCs, pDCs display a distinct set of chemokine and Toll-like receptors [2-4]. In response to viruses and CpG DNA, pDCs become activated to produce IFN-α and their APC function is enhanced [5-8]. While pDCs were first demonstrated in the T-cell areas of lymph nodes [5,9], precursors of this DC population have been isolated from several sources, including normal peripheral blood (PB), thymus, fetal liver, and bone marrow [10]. Although they do not reside in normal peripheral tissues, pDCs have been shown to infiltrate certain inflamed tissues and tumor sites, including the skin in psoriasis and lupus, the cerebrospinal fluid in multiple sclerosis, and melanoma and ovarian carcinoma [11-15]. While pDCs play an important effector role in viral disease, being the major producers of IFN-α and having a primary role in innate immunity, there is also evidence that they may play an immunoregulatory role, through the induction of Th2 (T helper 2)-type cytokines [9,16-18].

The synovial autoimmune reaction of rheumatoid arthritis (RA) is characterized by lymphocyte, macrophage, and DC infiltration that can progress to the development of lymphoid tissue in established disease [19-21]. DCs are likely to contribute to the formation and maintenance of such organized lymphoid tissue and antigen presentation in RA and other autoimmune lesions [22-24]. We have previously shown that the effector site in RA synovial tissue is enriched in differentiated myeloid DCs, which express CD33, CD11c, MHC and costimulatory molecules, and nuclear RelB [21,25]. Translocation of RelB to the nucleus of myeloid DCs is associated with APC function, particularly through increased expression of MHC molecules CD86 and CD40 [26].

The proinflammatory cytokines TNF-α and IL-1β are key contributors to the inflammatory cytokine cascade in RA [27,28]. This relates to a number of actions, but activation of the endothelium by TNF-α is particularly important in cellular recruitment to the synovium [29-31]. Since RA is characterized by endothelial activation, leukocyte recruitment, and the development of high endothelial venules, we hypothesized that pDCs would be enriched in inflamed but not normal synovium. Since the functional role of pDCs in disease pathogenesis is only partly understood, we also wished to address whether these cells represent a population distinct from the described nuclear RelB^+ ^synovial DCs, and whether they may contribute as APCs or inflammatory cells in RA [21].

## Materials and methods

### Patients and controls

Thirty patients who fulfilled the American College of Rheumatology criteria for RA were included [32]. Of these, 10 provided synovial fluid (SF) samples and 27 provided PB samples. Of the 30 patients, 80% were seropositive, 62% were female, and 73% were taking at least one disease-modifying antirheumatic drug or low-dose prednisone or both. Synovial tissue was obtained at arthroscopy from seven patients with RA, of whom three were untreated and four were taking at least one disease-modifying antirheumatic drug and low-dose prednisone. The duration of disease ranged from 0.5 to 18 years. In addition, we studied synovial tissue from four healthy individuals with nonspecific knee pain undergoing arthroscopy, one patient who had had psoriatic arthritis for 8 years, and one patient who had had ankylosing spondylitis for 30 years. Each patient with spondyloarthropathy was taking sulfasalazine. No patient in the study was taking biologics. Synovial tissue was provided by Dr Malcolm Smith (Repatriation Hospital, Adelaide, Australia). PB buffy coats prepared from 30 healthy donors were obtained from the Red Cross Blood Transfusion Service (Brisbane, QLD, Australia). The study was approved by the Research Ethics Committee of the Princess Alexandra Hospital.

### Culture medium and cell isolation

All cells were cultured in RPMI 1640 (Gibco, Life Technologies, Mulgrave, VIC, Australia) supplemented with 10% FCS (CSL Ltd, Parkville, VIC, Australia), 0.3 mg/ml L-glutamine (Trace Biosciences, Castle Hill, NSW, Australia), 0.12 mg/ml benzylpenicillin (CSL), and 10 μg/ml gentamicin (Delta West, Pharmacia and Upjohn, Spring Hill, QLD, Australia). The monoclonal antibodies used in this study include FITC, phycoerythrin (PE), and purified anti-CD11c, CD14-PerCP, PE, biotinylated and purified anti-CD123, CD86-FITC (all from BD Pharmingen, San Diego, CA, USA), BDCA2-FITC (Miltenyi Biotech, San Francisco, CA, USA), HLA-DR-biotin (Coulter Immunotech, Fullerton, CA, USA), CD40-FITC (Biolegend, San Diego, CA, USA), CD80-FITC (Cymbus Biotech, Chandlers Ford, Hants, UK), CD68 (Kp-1, DAKO, Carpinteria, CA, USA), RelB (C-19, Santa Cruz Biotech, Santa Cruz, CA, USA), and biotinylated *Ulex europaeus *agglutinin I (Vector Laboratories, Burlingame, CA, USA).

Mononuclear cells were prepared from normal or RA PB or RA SF by density gradient centrifugation over Ficoll-Paque (Pharmacia Biotech, Uppsala, Sweden) as described elsewhere [33]. T cells were purified from PB mononuclear cells by passing the cells over a nylon wool column, followed by immunomagnetic depletion of remaining monocytes, DCs, B cells, and NK (natural killer) cells using monoclonal antibodies against CD14, CD16, CD19, CD56, and HLA-DR (all from BD Pharmingen), followed by goat antimouse immunoglobulin magnetic beads, then passage through a strong magnetic field (MACS, Miltenyi Biotech), and collection of the unbound fraction. On analysis by flow cytometry, the unbound fraction routinely contained 95–98% CD3^+ ^T cells. DC-enriched non-T cells were produced by immunomagnetic depletion of T, B, and NK cells from non-T cells, by incubation with monoclonal antibodies against CD19, CD16, CD56, and CD3.

### Flow cytometric analysis and selection of cells by cell sorting

To enumerate CD123^+ ^and CD11c^+ ^subsets of DCs, mononuclear cells from normal PB or RA SF were stained for four-colour flow cytometry as described elsewhere [33], using monoclonal antibodies against CD14-PECy5, CD11c-FITC, CD123-PE, and HLA-DR-APC. Live CD14^-^HLA-DR^+ ^mononuclear cells were gated for analysis. Subset percentages are expressed as percentage of total mononuclear cells. Listmode data were analyzed using Winlist 2.0 software (Verity Software House, Topsham, ME, USA). For sorting, PB or SF DC-enriched non-T cells stained with the same four markers were sorted using the Moflo flow cytometer (DAKO), gating on CD14^-^HLA-DR^+ ^and either CD123^+^CD11c^- ^or CD11c^+^CD123^- ^cells, respectively. For phenotypic analysis, mononuclear cells from PB or SF were stained with CD14-PECy5, CD123-PE or CD11c-PE, HLA-DR-APC, and either a fourth monoclonal antibody or isotype control monoclonal antibody conjugated with FITC. DCs were gated as described above.

### Electron microscopy

Electron microscopy of freshly sorted cells was carried out as described elsewhere [5]. After fixation in 2.5% glutaraldehyde in phosphate-buffered saline, the cells were post-fixed with an aqueous solution of 1% OsO_4 _containing 1.5% K_4_Fe(CN)_6_. Subsequently, the specimens were dehydrated in an alcohol series and embedded into epon. Ultrathin sections (50 nm) were contrasted with lead citrate and uranyl acetate and studied with a CM100 electron microscope (Philips, Eindhoven, The Netherlands).

### Mixed lymphocyte reactions and cytokine analysis

Various numbers of sorted PB or RA SF DCs were incubated with 10^5 ^allogeneic PB T cells in triplicate wells for 5 days, as described elsewhere [33]. Supernatants were removed from some cultures and [^3^H]thymidine (1 μCi/well, ICN Biochemicals) was added to the remainder for the final 18 h. Cells were harvested onto glass-fiber filter mats and the incorporation of [^3^H]thymidine was determined by liquid scintillation spectroscopy (Packard Topcount, Packard Instrument Co, Meriden, CT, USA). IFN-γ, IL-10, and TNF-α were measured in supernatants by ELISA using OptEIA ELISA kits (BD Pharmingen).

### Immunohistochemistry

Frozen or paraffin-embedded sections of synovial tissue from patients with untreated active RA were obtained by arthroscopic biopsy and supplied by Malcolm Smith (Repatriation Hospital, Adelaide, Australia). Normal synovial tissue was obtained at arthroscopy from patients undergoing arthroscopy for nonspecific knee pain and in whom no abnormality was found. After fixation with acetone, sections were stained with anti-CD11c or anti-CD123 using an immunoperoxidase technique, and revealed with diaminobenzidine (brown). Frozen sections were double-stained with *U. europaeus *agglutinin I (Ulex), a lectin that specifically binds endothelial cells (fast red), and anti-CD123 (brown), using a double, immunoperoxidase–immunoalkaline phosphatase technique as described elsewhere [34]. Formalin-fixed paraffin-embedded sections were antigen-retrieved in 10 mM citrate buffer at pH6 in an autoclave, then stained with anti-CD123 (diaminobenzidine, brown) alone, or in combination with anti-RelB (BCIP, DAKO, purple). Sections were counterstained with hematoxylin except when they had been double-stained for CD123 and RelB and were photographed using a transmitted-light microscope (Leitz Diaplan, Leica, Germany). To quantitate infiltration by CD123^+ ^DCs, the number of CD123^+^Ulex^- ^cells was counted in sections double-stained with CD123 and Ulex. Cells were counted in each of the entire sections from three patients and three normal controls at high power, and for each biopsy this number was corrected for the area of the section to obtain the number per mm^2^. To quantitate infiltration by CD11c^+ ^cells, the number of these cells was counted in three high-power fields of the synovial sublining in sections from three patients and three normal controls.

### Statistical analysis

Differences were analyzed using unpaired Student's *t*-tests.

## Results

### CD123^+ ^nuclear RelB^- ^DCs are located in perivascular regions of RA synovial tissue

**Figure 1:**
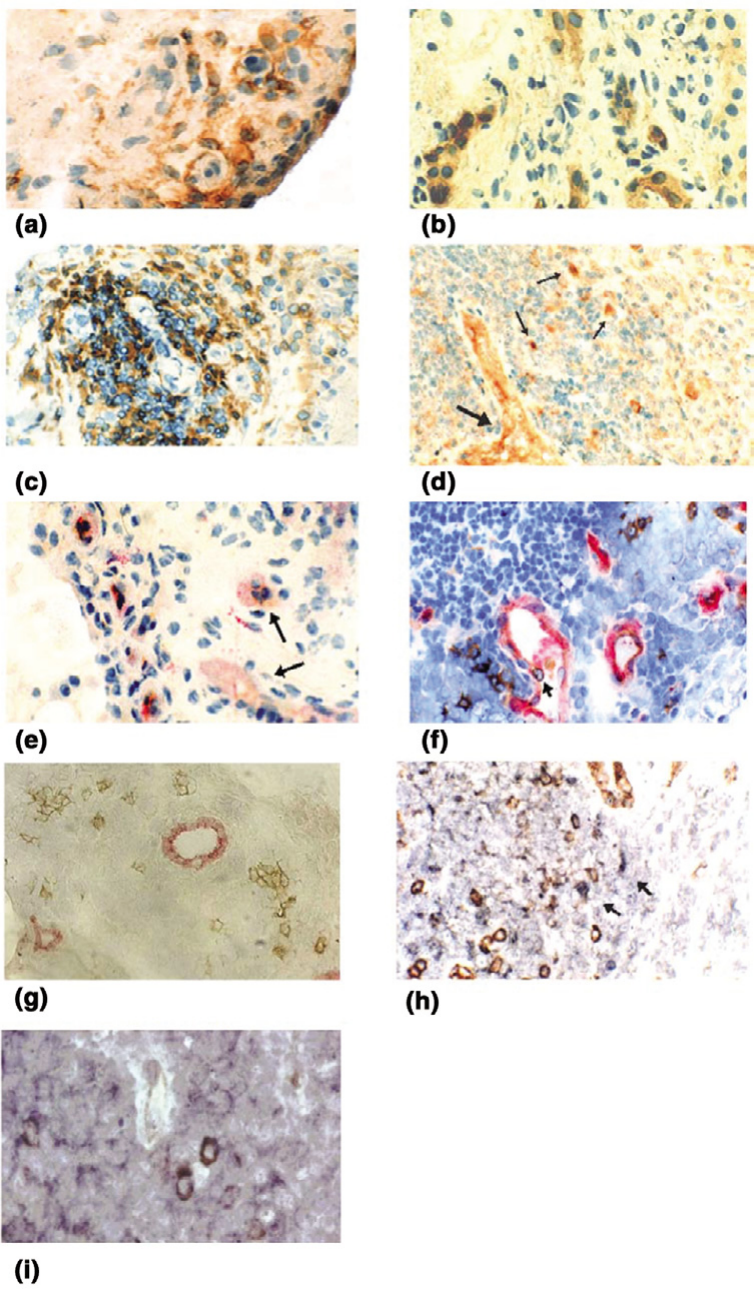
Nuclear RelB^-^CD123^+ ^plasmacytoid dendritic cells (pDCs) are located in close association with cells expressing tumor necrosis factor (TNF)-α in lymphoid aggregates of rheumatoid arthritis (RA) synovial tissue. Sections of frozen normal human synovial tissue **(a, b, e) **or synovial tissue from a patient with untreated active RA **(c, f, g) **or formalin-fixed sections from a patient with active RA **(d, h, i) **were stained with anti-CD11c (brown, **a, c**), anti-CD123 (brown, **b, d**), or anti-TNF-α (brown, **g**) using an immunoperoxidase technique. For double staining, sections were stained with Ulex (red) and anti-CD123 (brown, **e, f, g**) or with RelB (purple) and CD123 (brown, **h, i**) using a double, immunoperoxidase–immunoalkaline phosphatase technique. All sections were counterstained with hematoxylin (blue) except h and i, in which the nucleus of CD123^+ ^cells appears as a hole. The thick arrow in d identifies a blood vessel. Thin arrows denote representative CD123^+ ^perivascular DCs (d), representative double-stained vessels (e), a CD123^+ ^cell within a blood vessel (f), and nuclei stained by RelB (h). Data are representative of at least three separate RA donors in individual experiments. Scale bars represent 20 μm.

We have previously shown that synovial tissue in RA and spondyloarthropathy is enriched in differentiated myeloid DCs that express CD33, CD11c, MHC class II, costimulatory molecules, and nuclear RelB [21,25]. Translocation of RelB to the nucleus is associated with maturation and APC function of myeloid DCs [26]. These nuclear RelB^+ ^DCs are absent in normal synovial tissue and are rare in RA SF [21,23]. To determine whether RA synovial tissue was infiltrated by CD123^+ ^pDCs in addition to CD11c^+ ^myeloid cells, frozen synovial tissue sections, either normal or from patients with RA or spondyloarthropathy, were stained with CD11c or CD123. CD11c^+ ^cells were found both in the lining layer and adjacent to vessels in the sublining of normal synovial tissue. In contrast, CD123 only stained endothelial cells in the normal tissue (Fig. 1a,1b). In RA synovial tissue, CD11c again stained cells adjacent to vessels, now within lymphoid aggregates in the sublining. A population of CD123^+ ^cells with dendritic appearance was also stained adjacent to CD123^+ ^blood vessels in RA (Fig. 1c,1d,). Cells expressing TNF-α in RA synovial tissue were found in a similar location in serial sections (data not shown), as demonstrated previously [35]. To confirm the perivascular CD123^+ ^cells in synovial tissue, normal and RA synovial tissue were double-stained with the endothelial cell marker Ulex agglutinin (red) and with CD123 (brown). Whereas all CD123^+ ^structures in normal synovial tissue colocalized with Ulex agglutinin (orange), single-stained CD123^+ ^cells (brown) were located in perivascular lymphoid aggregates and within the lumen of occasional blood vessels in RA synovial tissue (Fig. 1e,1f). These CD123^+ ^cells are similar in appearance to those previously demonstrated as CD123^+ ^pDCs in human tonsil, in that they are smaller than CD11c^+ ^myeloid DCs, with shorter dendritic processes, and cell clusters gave the appearance of locally proliferating cells (Fig. 1g) [5,36]. While some macrophages can express CD123, there was no colocalization in synovial tissue of CD123 and CD68 (data not shown). However, aside from the dendritic morphology, we cannot exclude that some of the CD123^+ ^cells stained are mast cells [37]. To determine whether CD123^+ ^cells in synovial tissue were also nuclear RelB^+^, formalin-fixed tissue was double-stained for RelB and CD123 without hematoxylin counterstaining. No CD123^+ ^cells had translocated RelB to the nucleus, although some expressed cytoplasmic RelB (Fig. 1h,1i). In contrast, nuclear staining of RelB was evident in adjacent CD123^- ^cells (Fig. 1h, arrows). All patients with RA showed similar infiltration by pDCs and no differences in the cell numbers or location were noted between patients with RA or spondyloarthropathy (data not shown).

**Figure 2:**
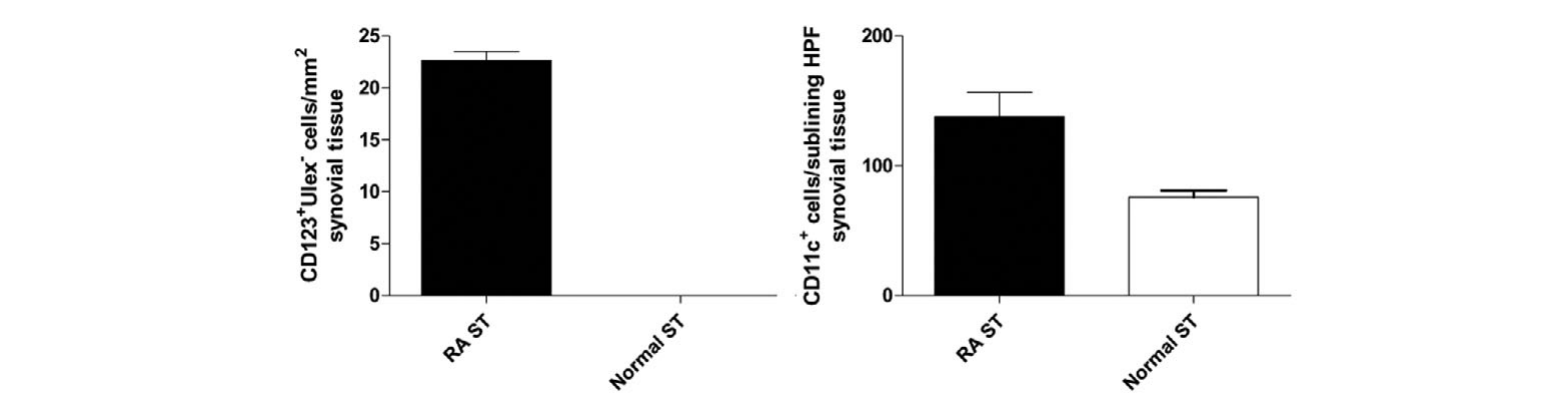
CD123^+ ^dendritic cells (DCs) and CD11c^+ ^cells are enriched in rheumatoid arthritis (RA) synovial tissue (ST). CD123^+ ^DCs were quantitated by counting the number of CD123^+^Ulex^- ^cells in sections double-stained with CD123 and Ulex. Cells were counted in each of the entire sections from three patients and three normal controls at high power, and for each biopsy this number was corrected for the area of the section to obtain the number/mm^2^. To quantitate CD11c^+ ^cellular infiltration, the number of CD11c^+ ^cells was counted in three high-power fields of the synovial sublining in sections from three patients and three normal controls. Data represent means ± standard error of the mean.

We quantitated pDCs in normal or RA synovial tissue by counting CD123^+^Ulex^- ^cells in synovial tissue sections from patients with RA or normal controls stained with CD123 and Ulex as shown in Fig. 1. Whereas no pDCs infiltrated the normal tissue, approximately 22 pDCs per mm^2 ^were identified within the RA tissue (Fig. 2). This number is similar to the number of nuclear RelB^+ ^differentiated DCs identified previously in RA synovial tissue [38]. In contrast, CD11c^+ ^cells infiltrated both normal and RA synovial tissue, with significantly larger numbers in RA (*P *< 0.05) (Fig. 2). We conclude that the CD123^+ ^cell population is most likely a pDC population that infiltrates RA and spondyloarthropathy but not normal synovial tissue and that it is distinct from the described nuclear RelB^+ ^DCs [21,36,39]. CD11c^+ ^cells comprise immature and differentiated myeloid DCs as well as monocytes [1,34]. Differentiated nuclear RelB^+ ^DCs are found within the CD11c^+ ^DC population in RA and other inflammatory arthritides but not in normal synovial tissue [21].

### CD11c^+ ^and CD123^+ ^DCs in RA SF

**Figure 3:**
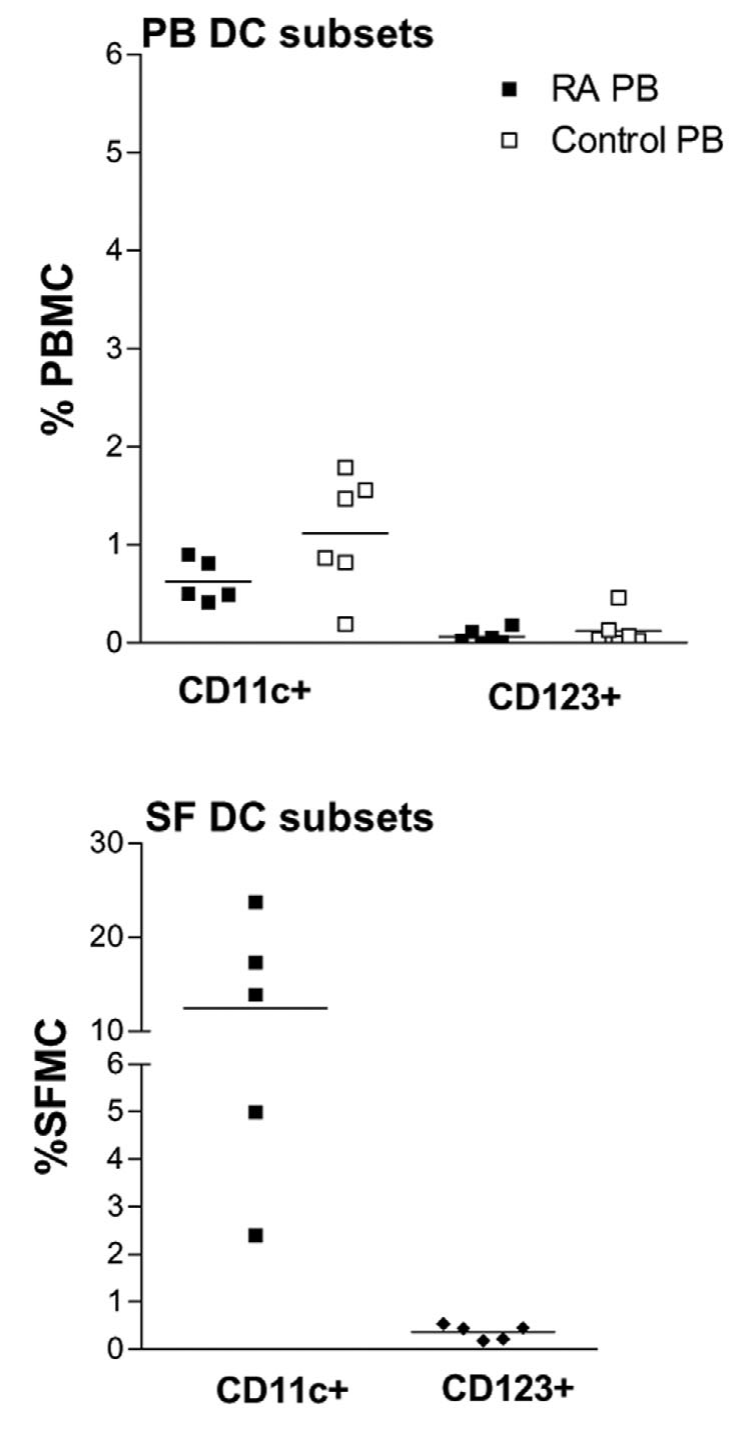
CD11c^+ ^dendritic cells (DCs) and CD123^+ ^DCs are present in rheumatoid arthritis (RA) synovial fluid (SF). After purification of mononuclear cells from either normal or RA peripheral blood (PB) or RA SF, cells were stained with CD123-PE, CD11c FITC, CD14-PECy5, and HLA-DR-APC. Live HLA-DR^+^CD14^- ^mononuclear cells were gated; polymorphonuclear cells were excluded on the basis of forward and side light-scatter. Scatter plots depict percentage of total PB or SF cells expressing the indicated markers for all donors. APC, antigen-presenting cell; PBMC, peripheral blood mononuclear cells; PE, phycoerythrin; SFMC, synovial fluid mononuclear cells.

Workers in our laboratory have previously shown that RA SF is enriched in CD11c^+^CD33^bright^CD14^- ^myeloid DCs with efficient APC function [25,40]. However, when freshly isolated, only a small proportion of SF CD33^bright^CD14^- ^DCs have translocated RelB to the nucleus. RA and normal PB mononuclear cells contain similar proportions of CD33^bright^CD14^- ^DCs [25]. To examine plasmacytoid and myeloid DCs in parallel, we compared RA SF with RA and healthy, control PB for the proportion of CD123^+ ^and CD11c^+ ^HLA-DR^+^CD14^- ^DCs. After purification of mononuclear cells from either normal or RA PB or RA SF by gradient centrifugation, cells were stained with CD123-PE, CD11c-FITC, CD14-PECy5, and HLA-DR-APC. Polymorphonuclear cells were excluded on the basis of forward and side light-scatter. Since basophils and monocytes can also express CD123, potential CD123^+ ^non-DCs were excluded by gating CD14^-^HLA-DR^+ ^cells [10]. By four-color analysis, CD14^-^HLA-DR^+^CD123^+ ^and CD11c^+ ^DC populations could be distinguished (Fig. 3). The percentages of CD123^+^CD11c^- ^pDCs in RA PB and normal PB were low and did not differ from each other. This observation contrasts with the reduction in pDCs observed in blood from patients with systemic lupus erythematosus [41]. CD11c^+^CD123^- ^myeloid DCs were more common than CD123^+ ^DCs in patient and control blood (*P *< 0.005), in keeping with previous studies of normal PB [1]. RA SF contained a significantly greater percentage of CD11c^+ ^DCs than normal or RA PB (*P *< 0.005) – in accord with previous studies using the markers CD33 and CD14 [40]. The proportion of CD11c+ DCs in RA SF was higher than that of RA SF CD123+ DCs (*P *< 0.05). Although the difference was small, the percentage of CD123^+ ^DCs in RA SF was higher than in RA or control PB (*P *< 0.05). The data show that CD123^+ ^DCs are present in RA SF, and that the ratio of CD11c^+ ^to CD123^+ ^DCs is similar in RA SF to that in normal or RA PB (approximately 10:1). In RA synovial tissue, mature myeloid nuclear RelB^+ ^and CD123^+ ^DCs have infiltrated perivascular lymphoid aggregates in similar numbers. Previously, similar numbers of immature and mature myeloid DCs were identified in RA synovial tissue [42]. Thus pDCs make up about 30% of DCs within RA synovial tissue. The present and previously published data, taken together, show that both pDCs and myeloid DCs are recruited to RA synovium, with an enrichment of pDCs in synovial tissue relative to blood or SF.

### CD123^+ ^PB DCs are immature whereas SF pDCs show signs of activation

**Figure 4:**
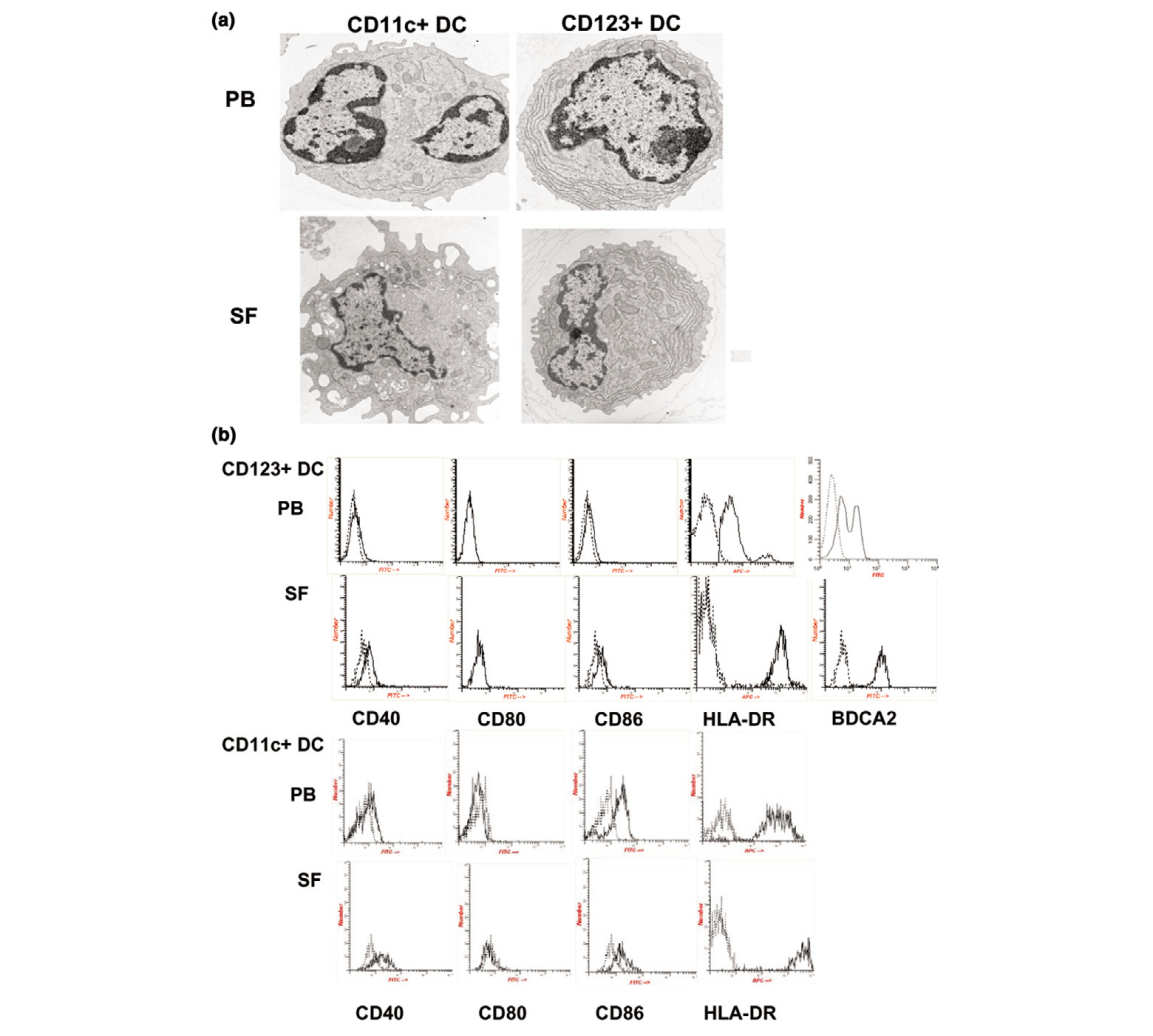
Morphology and cell-surface phenotype of dendritic cell (DC) subpopulations. **(a) **Freshly isolated CD11c^+^CD123^- ^DCs and CD123^+^CD11c^- ^DCs were sorted from either normal peripheral blood (PB) or rheumatoid arthritis (RA) synovial fluid (SF) according to the gating strategy outlined in the legend to Fig. 3 and were then prepared for transmission electron microscopy. Micrographs are representative of three separate donors. Scale bars represent 20 μm. **(b) **Normal PB or RA SF mononuclear cells were stained with CD123-PE, CD14-PECy5, HLA-DR-APC, and either isotype control or the depicted marker labelled with FITC. Live HLA-DR^+^CD14^-^CD123^+ ^cells or HLA-DR^+^CD14^-^CD11c^+ ^cells were gated; polymorphonuclear cells were excluded on the basis of forward and side light-scatter. The expression of isotype control (dotted lines) and indicated markers (continuous lines) by the gated cells are depicted. Data are from three donors of normal PB or RA SF. APC, antigen-presenting cell; PE, phycoerythrin.

In normal PB, pDCs circulate as precursors with the potential for recruitment into tissues in response to chemokines [2,43]. These precursors exhibit a characteristic plasmacytoid morphology on electron microscopy, a cell-surface phenotype characterized by expression of the BDCA2 antigen, by low levels of costimulatory molecule expression, and by the potential for IFN-α production in response to viral or immunostimulatory CpG DNA motifs [1]. We therefore analyzed the characteristics of sorted RA SF CD123^+ ^DCs and compared them with control PB CD123^+ ^DCs. On electron microscopic examination, freshly sorted PB and SF CD123^+ ^DCs appeared similar, with a smooth surface and abundant rough endoplasmic reticulum in the cytoplasm. The nucleus was nonlobulated and abundant in euchromatin and contained a distinct nucleolus (Fig. 4). CD11c^+ ^PB DCs were morphologically distinct from the CD123^+ ^pDCs, with a lobulated nucleus and some phagocytic vesicles. CD11c^+ ^DCs from SF showed more membrane ruffling and phagocytic activity than those from PB (Fig. 4). Thus SF CD123^+ ^DCs morphologically resemble CD123^+ ^DCs in PB, whereas CD11c^+ ^SF DCs display a greater level of ruffling and phagocytic activity, consistent with their enhanced level of activation, than CD11c^+ ^circulating precursors [21].

On four-color flow cytometric analysis, gated RA SF CD14^-^HLA-DR^+^CD123^+ ^DCs expressed low levels of CD40, CD80, and CD86. All or the majority of SF CD123^+ ^DCs expressed the BDCA2 marker of immature pDC precursors [1]. This cell-surface phenotype closely resembles that of control PB CD123^+ ^DC precursors, although BDCA2 was consistently expressed at high levels only by a subset of CD123^+ ^HLA-DR^+ ^cells in PB (Fig. 4b). No PB or SF cells expressed the DC differentiation marker CD83 (data not shown). However, SF CD123^+ ^and CD11c^+ ^DCs expressed higher levels of cell-surface HLA-DR than the corresponding cells in PB, suggesting some cellular activation within the SF environment [5,10,44]. Thus CD123^+ ^pDCs comprise a small proportion of RA SF mononuclear cells, which are predominantly immature but show some evidence of activation *in situ*. These observations regarding phenotype and PB and SF numbers are consistent with findings in two recent studies [39,45].

### CD123^+ ^and CD11c^+ ^SF DCs are efficient APCs

**Figure 5:**
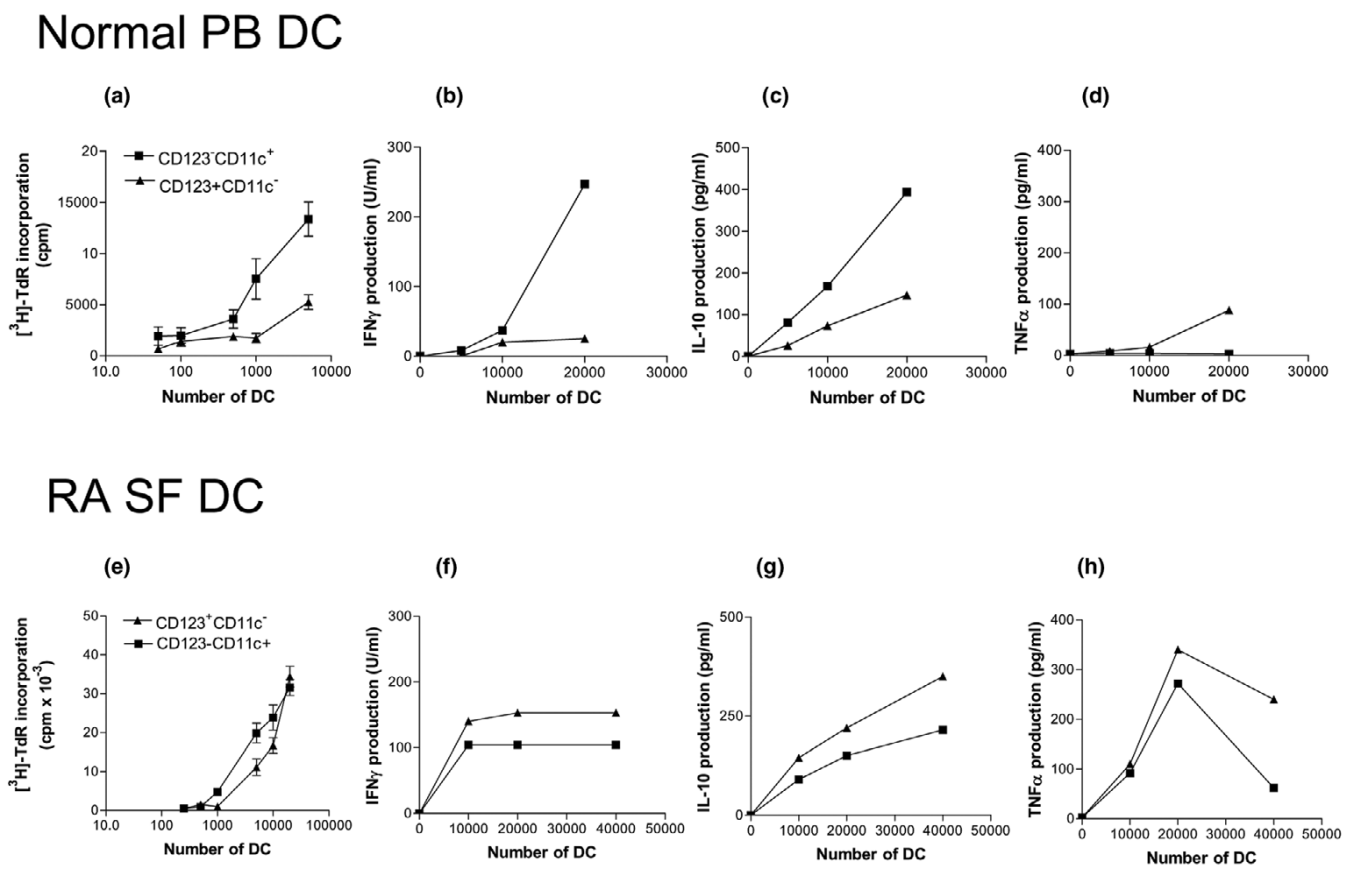
CD123^+ ^and CD11c^+ ^synovial fluid (SF) dendritic cells (DCs) are efficient antigen-presenting cells (APCs) and induce secretion of tumor necrosis factor α. Freshly sorted CD123^+^CD11c^- ^or CD11c^+^CD123^- ^DCs from either normal peripheral blood (PB) **(a–d) **or rheumatoid arthritis (RA) SF **(e–h) **were incubated with 10^5 ^purified normal allogeneic T cells. T-cell proliferation was measured by [^3^H]thymidine incorporation of triplicate wells after 60 hours (a, e). Unstimulated T-cell proliferation was routinely <500 cpm. Data represent means ± standard error of the mean of triplicate wells and are from three individual PB and SF donors. The concentrations of IFNγ, IL-10, and tumor necrosis factor (TNF)-α were measured in supernatants of allogeneic mixed lymphocyte reactions stimulated by sorted PB **(b–d) **or SF DCs **(f–h)**. Data are means of duplicate samples and are from two separate SF donors.

We have previously shown that freshly isolated CD33^bright^CD14^-^CD11c^+ ^SF DCs efficiently stimulate resting T cells in allogeneic mixed lymphocyte reactions [21]. In contrast, whereas freshly isolated CD11c^+ ^PB DCs are efficient APCs in mixed lymphocyte reactions, CD123^+ ^PB DCs usually require prior activation in the presence of IL-3 and CD154 for acquisition of APC function in this assay. To analyse the functional capability of RA SF DCs, CD11c^+ ^and CD123^+ ^DCs were sorted from either normal PB or RA SF and incubated with freshly isolated normal allogeneic PB T cells. Freshly isolated PB CD11c^+ ^but not CD123^+ ^DCs efficiently stimulated allogeneic T-cell proliferation and IFN-γ and IL-10 production in mixed lymphocyte reactions. Addition of IL-3 made no difference to the T-cell proliferation in response to CD123^+ ^DCs (data not shown), suggesting that death of the APCs was not responsible. In contrast, both freshly isolated CD11c^+ ^and CD123^+ ^SF DCs efficiently stimulated proliferation and IFN-γ and IL-10 production by resting normal allogeneic T cells (Fig. 5). A recent study demonstrated the capacity of RA SF to inhibit pDC differentiation *in vitro *[39]. The current studies are consistent, in that SF pDCs showed only some evidence of activation *in situ*, but once incubated in mixed lymphocyte reactions in the absence of SF they displayed enhanced APC function relative to that of PB pDCs. Whereas stimulation of mixed lymphocyte reactions either by CD11c^+ ^or by CD123^+ ^PB DCs resulted in little TNF-α production, stimulation by either of these DCs from RA SF resulted in high levels of TNF-α secretion (Fig. 5). The data indicate that pDCs have the capacity for enhanced APC function relative to PB pDCs once removed from the RA SF environment. Furthermore, at the time of antigen presentation by SF DCs to T cells, production of a number of cytokines by either T cells or DCs may be stimulated, including TNF-α, and this appears to be a characteristic of RA synovial DCs rather than the subtype of stimulating DCs.

## Discussion

Ongoing inflammation in RA involves positive feedback loops between activated T cells, B cells, DCs, macrophages, and their products, with destructive consequences for parenchymal cells. Clinical and animal data indicate that effector-site DCs play an important proinflammatory role in the perpetuation of autoimmune disease and contribute to the lymph-node-like organization of that tissue [22,46]. This role may be effected by local antigen presentation to CD4^+ ^and CD8^+ ^effector cells, but DC cytokine and chemokine secretion are also important [47,48]. TNF-α and IL-1β are important downstream proinflammatory and destructive cytokines in RA for somatic cells, whose release is promoted by activation of macrophages. IL-10 is highly expressed in RA, and IFN-γ is an important T-cell effector cytokine [49,50].

In the current studies, we show that, in addition to the previously described population of nuclear RelB^+ ^DCs, a further population of nuclear RelB^-^CD123^+ ^pDCs is located in perivascular regions of RA but not normal synovial tissue sublining. Moreover, pDCs were located within blood vessels, and both DC populations were observed in perivascular areas in which cells producing TNF-α were colocated [35]. Adherence of CD123^+ ^and CD11c^+ ^DCs to TNF-α-activated endothelium was higher than to resting endothelium *in vitro *(data not shown). TNF-α plays an important role in the recruitment of other leukocytes to RA synovial tissue [29], and this most likely pertains to the recruitment of pDCs to RA but not normal synovial tissue through expression of adhesion molecules such as intercellular adhesion molecule (ICAM)-1, CD62-E, and CD62-P and interaction with their ligands on pDCs [9,11,51-54]. Furthermore, TNF-α up-regulates synthesis of chemokines by endothelial cells [55]. During experimentally elicited allergic rhinitis, CD123^+^HLA-DR^+ ^pDCs have been shown to be recruited to human nasal mucosa [11].

The gene for MxA is specifically induced by IFN-α and therefore identifies a population of activated pDCs. In contrast, BDCA2 is a marker of immature pDCs. MxA^+ ^pDCs have previously been demonstrated in involved lupus skin and inflamed tonsil [13]. In RA synovial tissue, BDCA2 was shown to stain fewer cells than CD123 or MxA, suggesting differentiation *in situ *of a large proportion of pDCs into cells with a capacity for production of IFN-α and other cytokines. Together, the current and previous studies demonstrate recruitment of pDCs to normal lymphoid organs as well as inflammatory sites, with local differentiation, but no recruitment to normal peripheral tissues. In contrast, CD11c^+ ^myeloid precursors populate normal resting tissues, as shown here, but additional CD11c^+ ^myeloid cellular recruitment takes place at inflammatory sites, where RelB nuclear translocation takes place [21,56]. We have previously shown that, like synovial pDCs, CD123^+ ^DCs in the T-cell area of human tonsil are also nuclear RelB^- ^[23]. The data suggest either that activation of pDCs is not associated with nuclear translocation and transcriptional activity of RelB or that conditions in tonsil and synovium do not induce sufficient RelB translocation for detection by immunohistochemistry [26,57]. As preliminary studies *in vitro *demonstrate induction of RelB in PB pDCs after stimulation with lipopolysaccharide and CpG, and reduced production of IFN-α by pDCs in RelB-deficient mice, it is likely that RelB activation does accompany pDC activation. However, RelB translocation might be quantitatively reduced or RelB might be more rapidly degraded in the nucleus of pDCs than of myeloid DCs in inflamed tissues [58].

Of relevance to the RA inflammatory lesion, stimulation of blood pDC precursors with signals including CD154, influenza virus, or CpG oligonucleotides induces production of large amounts of cytokines, including IFN-α, IFN-β, and TNF-α; induction of DC differentiation; and stimulation of APC function [3,39,44,59]. Although inhibitory effects of SF on DC function, and thus on T-cell proliferation and cytokine production, are confirmed here [21,39,60], factors in the RA SF environment, such as IL-3 and CD154, may be sufficient to precondition the SF pDCs for efficient APC function *ex vivo *[36,44]. IFN-γ, IL-10, and TNF-α were produced in mixed lymphocyte reactions stimulated by myeloid or pDCs derived from SF but not PB, potentially by DCs or by T cells or both. In the tissue, as a result of antigen presentation by myeloid DCs or pDCs, key effector cytokines may be produced in perivascular areas in RA, located strategically close to endothelial cells, as well as incoming leukocytes. It is not known whether pDCs are capable, like myeloid DCs, of migration from synovial tissue to draining lymph nodes. However, it seems probable that pDCs conditioned by local IL-3 and CD154, or even viral or bacterial products transported to the synovium, predominantly play local proinflammatory and antigen-presenting roles, through secretion of cytokines such as IFN-α and possibly TNF-α [61,62].

## Conclusion

pDCs are recruited to RA synovial tissue and comprise an APC population distinct from the previously described nuclear RelB^+ ^synovial DCs. The APC function of pDCs is greater in SF than in PB. Activated pDCs and interacting T cells may contribute significantly to the inflammatory environment in RA.

## Abbreviations

APC = antigen-presenting cell; DC = dendritic cell; ELISA = enzyme-linked immunosorbent assay; FCS = fetal calf serum; FITC = fluorescein isothiocyanate; IFN = interferon; IL = interleukin; NK = natural killer; PB = peripheral blood; pDC = plasmacytoid DC; PE = phycoerythrin; RA = rheumatoid arthritis; SF = synovial fluid; TNF = tumor necrosis factor.

## Competing interests

The author(s) declare that they have no competing interests.

## Authors' contributions

LC, RT, LF, and PP conceived the experiments and LC, AB, LS, JP, and LF carried them out. LC, RT, and LF wrote the manuscript.
